# Prevalence and factors associated with musculoskeletal disorders and rheumatic diseases in indigenous Maya-Yucateco people: a cross-sectional community-based study

**DOI:** 10.1007/s10067-015-3085-9

**Published:** 2015-10-05

**Authors:** I. Peláez-Ballestas, J. Alvarez-Nemegyei, A. Loyola-Sánchez, M. L. Escudero

**Affiliations:** Rheumatology Department, General Hospital of Mexico “Eduardo Liceaga”, Dr. Balmis 148, Delegación Cuauhtémoc, C.P. 06726 Mexico City, Mexico; Regional High Specialty Hospital, Mérida, Yucatan Mexico; School of Rehabilitation Science, McMaster University, Hamilton, Canada; Universidad Autónoma Metropolitana, Mexico City, Mexico

**Keywords:** COPCORD, Indigenous community, Maya-Yucateco, Prevalence, Rheumatic diseases

## Abstract

This study aimed to estimate the prevalence of musculoskeletal disorders and rheumatic diseases in indigenous Maya-Yucateco communities using Community-Oriented Program for Control of Rheumatic Diseases (COPCORD) methodology. The study population comprised subjects aged ≥18 years from 11 communities in the municipality of Chankom, Yucatan. An analytical cross-sectional study was performed, and a census was used. Subjects positive for musculoskeletal (MSK) pain were examined by trained physicians. A total of 1523 community members were interviewed. The mean age was 45.2 years (standard deviation (SD) 17.9), and 917 (60.2 %) were women. Overall, 592 individuals (38.8 %; 95 % CI 36.3–41.3 %) had experienced MSK pain in the last 7 days. The pain intensity was reported as “strong” to “severe” in 43.4 %. The diagnoses were rheumatic regional pain syndromes in 165 (10.8 %; 95 % CI 9.4–12.5), low back pain in 153 (10.0 %; 95 % CI 8.5–11.6), osteoarthritis in 144 (9.4 %; 95 % CI 8.0–11.0), fibromyalgia in 35 (2.2 %; 95 % CI 1.6–3.1), rheumatoid arthritis in 17 (1.1 %; 95 % CI 0.6–1.7), undifferentiated arthritis in 8 (0.5 %; 95 % CI 0.2–0.8), and gout in 1 (0.06 %; 95 % CI 0.001–0.3). Older age, being female, disability, and physically demanding work were associated with a greater likelihood of having a rheumatic disease. In conclusion, MSK pain and rheumatic diseases were highly prevalent. The high impact of rheumatic diseases on daily activities in this indigenous population suggests the need to organize culturally-sensitive community interventions for the prevention of disabilities caused by MSK disorders and diseases.

## Introduction

Despite large variations in prevalence and risk factors, rheumatic diseases are a threat to public health worldwide, but all the more so in developing countries [1–3]. Considering the differential impact of rheumatic diseases in these countries, the International League of Associations for Rheumatology (ILAR), together with the World Health Organization (WHO), put forward the Community-Oriented Program for Control of Rheumatic Diseases (COPCORD) aimed at recognizing, preventing, and controlling rheumatic diseases. The program is also used to generate reliable epidemiological data from communities with limited resources [4–6].

Mexico has the largest indigenous population among the Latin American countries, a fact that is reflected in Mexico’s legal self-definition as a multicultural nation [7]. The National Commission for the Development of Indigenous Peoples (NCDIP or *Comisión Nacional para el Desarrollo de los Pueblos Indígenas* (CDI)) estimates that the indigenous population in Mexico is close to 12 million, which is roughly 11 % of the total population. Mexican indigenous communities that have managed to preserve their identity and language are characterized by high levels of underdevelopment and social marginalization which stem not only from unequal access to public resources but also from the discrimination and exclusion to which they have been exposed [8, 9].

There is a dearth of publications on the epidemiological impact and determinants of rheumatic diseases in Latin American indigenous populations. Given this paucity of information, it is difficult to develop and implement specific interventions to reduce the burden imposed by rheumatic diseases on these highly socially vulnerable populations. The main objective of the GLADERPO group (*Grupo Latino-Americano de estudio De Enfermedades Reumáticas en Pueblos Originario* or Latin American Study Group on Rheumatic Diseases in Indigenous Peoples) is to conduct studies on indigenous populations in the region aimed at closing this information gap and developing culturally sensitive interventions. This study is part of the regional effort conducted by the GLADERPO group in the state of Yucatan, México.

COPCORD studies in Latin America, specifically in Mexico [10], Guatemala [11], Cuba [12], Peru [13], Venezuela [14], and Brazil [15] have been mainly performed in non-indigenous groups. Studies in Mexico have found that the presence of osteoarthritis (OA) is associated with living in extremely underdeveloped areas [16], while rheumatoid arthritis (RA) is associated with individuals that only speak an indigenous language [17]. Multicenter and multinational Latin American studies on systemic lupus erythematosus (GLADEL) [18] and RA (GLADAR) [19] have found significant differences between populations, particularly in terms of clinical and sociodemographic characteristics, age at disease onset, length of diagnostic delay, disease activity, and severity. Those studies have shown that the prevalence of inflammatory rheumatic diseases is higher in indigenous or socially disadvantaged populations [20, 21]. Although not without merit, these studies were based on hospital populations and focused on specific diseases.

Yucatan was chosen because it has the largest number of monolingual speakers, a high degree of social underdevelopment, and a high prevalence of RA [17]. The study had the following main objectives (using COPCORD research methodology): (1) to estimate the prevalence of musculoskeletal (MSK) disorders and rheumatic diseases; and (2) to determine the factors associated with such prevalence in adults from a Maya-Yucateco rural community.

## Materials and methods

### Study design

We conducted analytical cross-sectional community-based study using COPCORD methodology cross-culturally validated for the Maya-Yucateco population [22].

### Study population

We took a census of all 11 communities from the municipality of Chankom, Yucatan. All persons identified as indigenous and aged ≥18 years were invited to participate in the study. The municipality of Chankom is located in the state of Yucatan in southeastern Mexico. It consists of 11 communities and is classified as highly marginalized. The total population is 4464; of them, 3017 are ≥15 years of age, 3160 are bilingual, 565 are monolingual, 2392 inhabitants aged ≥15 years are literate, and 619 cannot read or write [8]. In addition, 3366 inhabitants of Chankom are reported to have “Seguro Popular” (a voluntary public health insurance targeted to low-income families without access to social security benefits; sources of funding comprise contributions from the federal government, the states, and affiliated families, the latter being defined on an income basis) [9], and 4412 inhabitants of Chankom were born there. The economically active population (persons who during the referenced period performed an economic activity) is 75.2 % [8].

Chankom was chosen based on the following characteristics: (1) the population is considered to be comprised of indigenous people by the NCDIP); (2) >90 % of the population speak an indigenous language; (3) according to the National Council for the Evaluation of Social Development Policy (Spanish: *Consejo Nacional de Evaluación de la Política de Desarrollo Social*, CONEVAL), the municipality has a high degree of social underdevelopment; and (4) the community wished to participate in the study [8].

The COPCORD questionnaire has 8 sections: (a) sociodemographic data, (b) self-reported comorbidities, (c) employment history, (d) MSK pain during the past 7 days, measured as pain intensity (0–10) and perceived severity (0–10), (e) MSK pain for any period of time in the past, (f) functional disability, (g) coping, and (h) help-seeking behavior [7].

### Survey

The methodology applied in each community was as follows: (1) standardized training of seven bilingual interviewers in administering the COPCORD questionnaire translated into the Maya-Yucateco language; (2) adjustment of the questionnaire to the field strategy; (3) validation of the questionnaire in four pilot tests [22]; (4) invitation to participate in the study during community assemblies and then individually during home visits; (5) obtaining signed consent forms in Maya-Yucateco and Spanish; (6) administering the COPCORD questionnaire to screen for MSK disorders, at home as a door-to-door survey. If needed, interviewers visited the same household as many as seven times to administer the questionnaire. Surveys were cross-checked by different interviewers during screening and later by the coordinators; (7) evaluation of mechanical stress (activities involving repeated handling objects >20 kg, standing for more than 30 min, and other physical activities such as bending); (8) administering the Health Assessment Questionnaire (HAQ-DI) validated for the Maya-Yucateco population [23] along with a socioeconomic questionnaire (level of education, monthly income, living conditions, access to health care, and material situation); (9) examination of positive cases—individuals with pain, stiffness and/or inflammation during the last 7 days or at some point in life—conducted by primary care physicians previously trained by a certified rheumatologist in MSK assessment; (10) examination of the probable rheumatic disease cases by a rheumatologist and a physiatrist to make a diagnosis based on the accepted classification criteria [24–31].

The study was conducted from February to December 2012. Patients with pain but no rheumatic disease were diagnosed and referred to secondary public health care for follow-up. The consultations were conducted in Maya-Yucateco language with the help of local trained medical interpreters. The data obtained in the interviews were collected and stored in a specially designed database for later analysis. Questionnaires with any missing data were excluded from the analysis.

### Statistical analysis

A descriptive analysis with measures of central tendency and dispersion for continuous variables was performed. Absolute and relative frequencies for categorical variables were estimated. An inferential analysis was initially performed by one-way and two-way analysis of variance (ANOVA) for continuous variables. We also used the chi-square test with Yates’s correction or Fisher’s exact test (according to the requirements) for categorical variables, with the diagnosis of a rheumatic disease and presence of any level of HAQ-DI disability as outcome variables. A logistic regression analysis was performed with forward steps, with diagnosis of a rheumatic disease as the outcome variable. The predictor variables in this analysis were grouped into three blocks: (1) socioeconomic variables: age, gender, education, monthly income, housing conditions, access to health care, material situation; (2) clinical variables: body mass index (BMI), self-reported comorbidity, characteristics of MSK pain; (3) physical function variables: HAQ-DI disability index results used as dichotomous variables, where 0 = no difficulty and 1 = any difficulty in performing activities ranging from mild difficulty to being unable to perform the activity in question. MSK biomechanical stress variables were also estimated.

The criterion for using the variables in the model was whether they were statistically significant in the univariate analysis or were biologically plausible. The level of significance was set at ≤0.05 (two-tailed test). Statistical analyses were performed using Stata *SE* version 11.0 for Mac.

### Ethical aspects

The study objective, strategy, and importance of information confidentiality were explained in detail to each participant. The study was approved by municipal authority, a local assembly of all participating communities, and the Ethics and Research Committees of the General Hospital of Mexico (Mexico City) and the Anahuac-Mayab University (Mérida, Yucatan, Mexico). Informed consent forms were signed in Maya-Yucateco or Spanish, with each participant receiving a copy. All subjects with any health problems detected during the study were referred to the appropriate health care professional.

## Results

A total of 1523 subjects (67.9 %) from 2242 people aged ≥18 years living in the selected community participated in the study. Table 1 shows the demographic and socioeconomic characteristics. Worthy of note are a predominance of female subjects, reliance on government-subsidized health care plans, and low income.

**Table 1:** Demographic and socioeconomic characteristics of the study population (*n* = 1523)

Variable	Result^a^
Women	917 (60.2)
Age (years); mean (SD)	45.2 (17.9)
Level education (years of formal education, *n* = 1515); mean (SD)	4.5 (3.6)
Body mass index (kg/m^2^, *n* = 1496); mean (SD)	28.1 (4.8)
Married	1188 (78.0)
Unemployed	68 (4.4)
Reason for unemployment (*n* = 65)	
Health problems	50 (76.9)
Work problems	6 (9.2)
Age	7 (10.8)
Retired	2 (3.0)
Monthly income in US dollars (year 2013) (*n* = 1504)	
<198.77	1412 (93.90)
198.77–397.55	72 (4.80)
397.56–795.10	17 (1.10)
795.11–1391.43	2 (0.13)
>1391.43	1 (0.07)
Type of health care (*n* = 1514)	
None	80 (5.3)
National Health Insurance “Seguro Popular”	1405 (92.8)
Total social covered	28 (1.8)
Private	1 (0.1)
Type of regularly seen health care professional: (*n* = 816)	
Medical doctors	390 (47.8)
Traditional alternative medicine (spiritualist, bone setter, herbalist, traditional healer, or chiropractor)	104 (12.7) 78 (bone setter)
Not seeking care	322 (39.5)
Human waste management *(n* = 1515)	
Toilet bowl	802 (52.9)
Outdoor defecation	454 (30.0)
Latrine	259 (17.1)

^a^Unless otherwise specified, values are depicted as *n* (%)

*SD* standard deviation

The most common work activities were housekeeping (826; 56.7 %), agriculture (425; 29.1 %), hand craftsmanship (67; 4.6 %), construction (18; 1.2 %), and independent work (24; 1.6 %). The patterns of biomechanical stress associated with the work activities are shown in Table 2. The vast majority of the economically active individuals performed activities involving frequent exposure to static and dynamic biomechanical stress.

**Table 2:** Work biomechanical stress patterns found in economically active individuals^a^ (*n* = 1454)

Variable	*n* (%)
Mechanical stress	388 (25.4)
Dynamic mechanical stress	424 (27.8)
Shaking hands	358 (23.9)
Pushing an object >20 kg (SI)	897 (60.0)
Handling loads >20 kg	974 (65.1)
Frequently going up or down stairs	723 (47.4)
Walking for over 30 min	1371 (90.0)
Frequently standing and sitting	1243 (81.6)
Static mechanical stress	878 (57.6)
Standing for over 30 min	1332 (87.4)
Bending down for over 30 min	911 (59.8)

^a^Refers to any activity that the respondent considers as part of the work activities (paid for in cash or in kind, e.g., agricultural or livestock product for self consumption)

The main self-reported comorbidities were alcoholism (537; 35.2 %), obesity (459; 30.1 %), anxiety (426; 27.9 %), depression (364; 23.9 %), blood hypertension (204; 13.3 %), gastritis (170; 11.1 %), smoking (148; 9.7 %), diabetes (149; 9.7 %), peripheral vascular disease (87; 5.7 %), and heart disease (67; 4.4 %). Overall, 55.3 % of the participants reported having a family member with a rheumatic disease.

MSK pain in the last 7 days (COPCORD-positive subjects) was reported in 592 (38.8 %; 95 % CI 36.3–41.3) individuals, of whom 198 (33.5 %) reported their pain as severe; 215/592 (36.2 % 95 % CI 32.4–40.3) reported pain associated with trauma. Historical pain was present in 779 (51.2 %; 95 % CI 48.6–53.7), of whom 240 (30.9 %) reported their discomfort as severe, and 199/779 (25.2 %) associated it with trauma. The most commonly used medications for treating MSK disorders were non-steroidal anti-inflammatory drugs (NSAIDs), (789/1034; 76.3 %), analgesics (137/1034; 13.2 %), and systemic glucocorticoids (77/1034; 7.4 %). When asked whether they had seen a doctor and received a diagnosis, 114/522 (21.8 %) answered affirmatively and 99 (86.8 %) replied that they were diagnosed with RA; 13 (11.4 %) said they had “rheumatism.”

Most common pain sites in the last 7 days and in the past were the knees (16.5 and 18.6 %), back (11.75 and 11.4 %), shoulders (11.4 and 11.5 %), hands (10.6 and 10.7 %), ankles (8.5 and 9.5 %), feet (7.7 and 7.2 %), and hips (7.5 and 6.7 %).

The prevalence of rheumatic diseases was 34.2 % (95 % CI 31.8–36.7 %). The most frequently diagnosed conditions were rheumatic regional pain syndromes (RRPS), low back pain, and OA (including localized and generalized OA). Table 3 shows the prevalence and 95 % CIs of rheumatic diseases. Two families with multiple cases of RA (0.3 %; 95 % CI 0.01–0.4) were found. We identified two concomitant diseases in 11/522 (21.2 %) patients, three diseases in 21 (4.0 %) patients, and four diseases in three (0.5 %) patients.

**Table 3:** Prevalence of rheumatic diseases and muscle-skeletal disorders

Rheumatic diseases	Cases	Prevalence (%)	95 % CI
Rheumatic regional pain syndrome^a^	165	10.8	9.4–12.5
Nonspecific low back pain	153	10.0	8.5–11.6
MSK disorders	146	9.5	8.1–11.1
Associated with infection	6	0.3	0.1–0.8
Associated with neurological disorders	19	1.2	0.7–1.9
Associated with vascular disorders	11	0.7	0.3–1.2
Associated with orthopedic disorders	24	1.5	1.0–2.3
Osteoarthritis^b^	144	9.4	8.0–11.0
Inflammatory back pain^c^	71	4.6	3.6–5.8
Fibromyalgia	35	2.2	1.6–3.1
Rheumatoid arthritis	17	1.1	0.6–1.7
Juvenile idiopathic arthritis	1	0.06	0.001–0.3
Nonspecific arthritis	8	0.5	0.2–0.8
Gout	1	0.06	0.001–0.3
Other^d^	86	1.5	0.9–2.2

^a^45 without specialized medical examination (by rheumatologist)

^b^Localized and generalized OA

^c^Back pain questionnaire was administered to 150 respondents, of which 71 met at least one criterion of 5 selected for the questionnaire. The most common criterion was improvement in pain level with exercise, followed by insidious pain (*n* = 47), morning stiffness >30 min (*n* = 37), night pain (*n* = 28), buttock pain (*n* = 26), swelling or stiffness in peripheral joints (*n* = 23)

^d^Neoplasms, surgical, metabolic, hematological

Table 4 shows the data for the self-reported physical limitation, HAQ-DI score, and degree of coping with pain in the COPCORD-positive subjects. In women, embroidery and sewing caused the most functional restrictions (5.1 %). In total, 107 (13.4 %) subjects reported that they had some physical limitation at the time of interview. Overall, 4.2 % of subjects revealed that they used assistive devices, cane being the most common (3.2 %).

**Table 4:** Functional impact of muscle-skeletal symptoms on COPCORD-positive subjects

	*n* (%)
Limitation (*n* = 779)	
Current physical limitation	107 (13.4)
Past limitation	179 (22.4)
Severity of discomfort (*n* = 794)	
No pain	39 (4.9)
Some pain	258 (32.5)
Mild pain	195 (24.6)
Severe pain	230 (28.9)
The most severe pain	72 (9.1)
Coping with discomfort (*n* = 814)	
No coping	119 (14.6)
Some coping	397 (48.8)
Good coping	284 (34.9)
Excellent coping	14 (1.7)

Univariate comparisons of sociodemographic and clinical factors of participants with rheumatic disease showed significant differences when compared to those without the disease. Participants with a confirmed rheumatic disease or MSK symptoms reported functional restrictions to standing on a stool, climbing stairs, bending, getting up, and doing housework (Table 5). There were exceptions in for sex, income, smoking, alcoholism (self-reported), job tenure, static stress and work activities involving spending more than 30 min bending, shaking hands, or pushing objects weighing >20 kg.

**Table 5:** Comparison between sociodemographic and clinical variables with the presence or absence of a diagnosed rheumatic disease (*n* = 1289)

Variable	Diagnostic of a rheumatic diseases^c^ *n* = 522	No rheumatic diseases^c^ *n* = 767	*p* value
Female gender	339 (64.9)	463 (60.3)	0.09
Mean age (SD)	52.2 (16.5)	39.6 (16.8)	<0.001
Marital status (married)	433 (82.9)	579 (74.8)	<0.001
Mean years of formal education (SD)	3.5 (3.3)	5.1 (3.6)	<0.001
Mean BMI (SD)	27.5 (4.6)	29.2 (5.0)	<0.001
Self-reported comorbidity			
Diabetes mellitus 2	74 (14.1)	49 (6.3)	<0.001
Blood hypertension	102 (19.5)	73 (9.5)	<0.001
Heart diseases	33 (6.3)	20 (2.6)	<0.001
Peripheral vascular diseases	43 (8.2)	24 (3.1)	<0.001
Gastritis	72 (13.7)	60 (7.8)	<0.001
Anxiety	213 (40.8)	125 (16.3)	<0.001
Depression	179 (34.2)	108 (14.0)	<0.001
Obesity^a^	187 (35.8)	211 (27.5)	0.02
Hyperlipidemia	52 (9.9)	16 (2.0)	<0.001
Mechanical stress	116 (22.2)	210 (27.3)	0.03
Dynamic mechanical stress	127 (24.3)	230 (29.9)	0.02
Standing for over 30 min	449 (86.1)	691 (91.0)	0.06
Work dynamic mechanical stress	127 (24.3)	230 (29.9)	0.02
Handling objects >20 kg	316 (60.6)	503 (66.2)	0.04
Frequently going up or down stairs	231 (44.3)	386 (50.8)	0.02
Walking for over 30 min	465 (89.2)	712 (93.8)	0.03
Functional capacity (HAQ-DI), median (IQR)^b^	0.1 (0–0.5)	0 (0)	<0.001

Chi-square test (dichotomous)

*ANOVA* one-way analysis of variance, *SD* standard deviation, *IQR* interquartile range

^a^Obesity: self-reported

^b^Kruskal Wallis test

^c^Unless otherwise specified, values are depicted as *n* (%)

A number of multiple logistic regression models were created to find the best model with a significant goodness-of-fit test (*p* = 0.01). The diagnosis of a rheumatic disease was associated with older age, being employed, work activities involving standing and walking for over 30 min, higher BMI, diabetes mellitus, anxiety, and depression (model 1). In the final model, the variables significantly associated with presence of a rheumatic disease were older age, being female, and high HAQ-DI score (model 2) (Table 6).

**Table 6:** Independent variables associated with the presence of rheumatic disease based on the multivariate regression analysis

	Model 1	Model 2	
Independent variables	OR (95 % CI)	OR (95 % CI)	*p*
Older age	1.04 (1.03–1.05)	1.03 (1.03–1.05)	<0.001
Being female	–	2.0 (1.7–3.3)	<0.001
Have job	7.6 (2.5–22.9)	–	<0.001
More dynamic mechanical stress	0.75 (0.58–0.98)	–	0.03
Standing for over 30 min	1.6 (1.0–2.7)	–	<0.001
Walking for over 30 min	0.56 (0.36–0.84)	–	0.006
HAQ-DI	55.9 (23.3–117.4)	13.0 (9.3–18.2)	<0.001
BMI	1.06 (1.03–1.09)	–	<0.001
Diabetes mellitus 2	1.7 (1.1–2.6)	–	<0.001
Anxiety	2.4 (1.7–3.3)	–	0.01
Depression	1.8 (1.3–2.5)	–	<0.001
Hyperlipidemia	3.2 (1.7–5.9)	–	<0.001

*OR* odds ratio. 95 % *CI* 95 % confidence interval

## Discussion

We found a high prevalence of MSK pain in the Maya-Yucateco indigenous population, with 38 % in the last 7 days and 51.2 % at some point in life. The most common diagnoses were RRPS, low back pain, OA, and fibromyalgia. Factors associated with the presence of any rheumatic disease were older age, being female, and chronic pain.

Pain has been described to vary across populations, and its perception may differ from one culture to another. We found higher prevalence of MSK pain in the last 7 days and at the least once in life compared to the open population in the state of Yucatan (22.8 and 26.3 %) [32], and in Aboriginal Australians (33 % and 22 %) [32], although lower than in indigenous communities of Guatemala (60.9 %) [11]. The prevalence of MSK pain in our study is higher than reported for the Maya-Yucateco participants with MSK symptoms (43 %), who reported pain ranging from strong to severe on a Likert scale; however, this result cannot be directly compared with other studies, which have used continuous visual analogue scales (ranging from 0 to 10) to measure pain intensity. However, we can draw comparisons with the visual analogue scale (VAS) used in some important studies for the purposes of the present study. Regarding the study in Aboriginal Australians, the results showed a VAS score of 53 [33], whereas in Yucatan, 17.5 % had a VAS score above 4.0 [32]. This brings to light the variability of prevalence and measuring pain intensity across different populations, as has been documented in another study on an indigenous population [9].

At the same time, the anatomical sites most affected by pain were largely similar. In our study, pain was more frequently seen in the knees and the back, which is in line with the study performed in Guatemala [11]. Conversely, back pain was predominant in Aboriginal Australians, followed by knee pain [25, 33]. This could be explained by the type of job performed by the participants in our study, which predominantly were activities such as housekeeping (56.7 %), farming (29.1 %), and to a lesser extent, construction work. This is similar to the Guatemalan indigenous population (40.2 %) [11] but differs from the Australian study (1.2 %) [25, 33].

The mean functional capacity (HAQ-DI) was higher in our study compared to the others COPCORD’s studies [11, 32, 33]. These findings demonstrate the level of impact of MSK disorders on the examined populations [33].

In our study, the prevalence of rheumatic diseases was 34.2 %, which is higher than the figure reported for the indigenous population of Guatemala (4.35 %) [11]. Interestingly, rheumatic diseases in the aboriginal population of Canada were the reason for utilizing the health care system in 21 %, being second only to diabetes mellitus type 2 [34]. The above observations lead us to propose that there is a need for qualified health care services to establish early diagnosis and to provide timely treatment.

In our study population, 48.9 % sought help in the biomedical health care system, and 10 % relied on the traditional medicine, which is similar to the reports on the aboriginal population in Canada [34]. It is interesting that a similar pattern of seeking health care was observed in two countries with different health systems, namely Canada with a publicly funded health care system and Mexico with a mixed and fragmented health care system. We can hypothesize that there are some sociocultural barriers in the health care process.

OA was higher (43 %) than reported for the indigenous communities in other countries [11, 33]. One possible explanation is the older age of the population surveyed in our study (45.2 years) compared with the studies in Guatemala [11] and Australia [33] at 36 and 35.2 years, respectively. There are other factors also associated with a higher prevalence of OA, such as activities involving significant physical effort. Between 60 and 80 % of participants reported doing highly physically demanding activities.

Low back pain had a prevalence of 10 %, which is higher than that in the Australian (3.8 %) [33] and Guatemalan (0.48 %) [11] studies. This may arise because 30.6 % of our study population were engaged in farming, compared with 7.5 % for the indigenous population in Guatemala [11], while 14.7 % of Aboriginal Australians performed work activities involving handling loads [33]. In our study, 60 and 65 % of the respondents reported that their work activities involved handling loads weighing >20 kg or pushing objects weighing >20 kg. These factors have been described as being associated with the presence of low back pain. Farming activities that typically involve handling and pushing loads weighting >20 kg could be responsible for the high prevalence of low back pain observed in the population in comparison with other indigenous populations in Guatemala and Australia [35].

The prevalence of RA in our study (1.1 %) is higher than that reported for other indigenous populations, comprising 0.8 % in the Guatemalan study [11] and 0 % in the Australian study [33]. Our prevalence of 1.1 % is lower than reported in a previous study performed in the same region (Yucatan) in an open population (2.8 %) [32]. One explanation may be that given the distribution of the sample, the population was predominantly comprised of city dwellers of mestizo descent. Another point to consider is migration to find better health care. Some study participants revealed that they had moved to cities within the same state to find treatment for RA. This was corroborated by home visits to some participants. This aspect has not systematically documented and remains a hypothesis to be further tested (health migration).

Fibromyalgia was found in 2.2 % of the population. This condition has not been reported in other studies on indigenous communities. In the open population study in Yucatan, the percentage was 0.2 % [32]. It is important to consider that one of the aspects associated with the presence of fibromyalgia is the need to cope with high levels of individual and social stress [36]. The prevalence of self-reported anxiety was 40 % in individuals diagnosed with a rheumatic disease and 16 % in subjects free of MSK disorders. In addition, the municipality of Chankom is classified as highly marginalized [23]. All these psychosocial factors could be related with the high prevalence of fibromyalgia observed in our study.

The prevalence of non-inflammatory rheumatic diseases was greater than that of inflammatory diseases. This is consistent with the results of the Guatemalan study [11] but differs from the findings of the study in the Aboriginal population in Canada, where inflammatory rheumatic diseases predominated [34]. Notably, much of the information referred to in the Canadian Aboriginal population study is the result of analyzing health care databases, which may entail a reference bias. The above does not rule out population variability in the presence of different rheumatic diseases. This differs from the findings in another study that reported a higher prevalence of inflammatory rheumatic diseases in minorities or socially disadvantaged populations [37].

Overall, 40 % of our participants reported not having sought or receiving medical attention for their MSK disorders, despite 25.8 % having or having had some severe physical limitation, 63.3 % had not been successful in coping with musculoskeletal disorders; all of the above contrasts with the report that 92.8 % had minimal social security. This is also in contrast with the findings of the Canadian study on an Aboriginal population, which was found to have a higher demand for health care than a non-aboriginal population [34].

Self-medication by NSAIDs was higher (76.3 %) than reported in another open population COPCORD study in Mexico (58.5 %). Risk of adverse events from an inadequate use of NSAIDs by a largely marginalized population with limited access to the biomedical health care needs to be considered, so health care education in indigenous communities would be a possible strategy to avoid complications due to NSAIDs abuse [38].

We can hypothesize that the remoteness of these communities from the reference health care centers, coupled with the lack of public transport and inability to speak other languages than Mayan (given that the health care centers in the state of Yucatan lack or have insufficient health care professionals speaking the language or culturally trained to provide a differential treatment) results in these communities being culturally marginalized.

Of note is the fact that subjects diagnosed with rheumatic diseases had lower education, lower BMI, and greater number of comorbidities, although smoking and alcoholism were less prevalent than in individuals without rheumatic diseases. These two last points are important in light of the association of smoking with rheumatic diseases, especially rheumatoid arthritis, and also of alcoholism being a factor associated with the prevalence of gout [33]. Smoking and alcoholism are highly prevalent among Aboriginal Australians. Other variables associated with the likelihood of a rheumatic disease were static mechanical stress (standing for over 30 min), physical labor, lower functional capacity, and current or past pain. These factors are very important in communities such as the one we examined, wherein work activities, the inhabitants are engaged in, are highly physically demanding (for housekeeping and farmers) and where pain is common and leads to physical limitations. Chronic pain needs to be detected in these populations, given that the subjects frequently learn to cope with such pain and the ensuing physical limitations. This coping strategy, coupled with the difficulties imposed by geographical, linguistic, and cultural barriers to early detection could lead to delays in seeking care [7, 8, 20].

This is one of the first attempts to conduct epidemiological studies on rheumatic diseases in indigenous populations using a standardized methodology that help to systematically identify subjects with rheumatic diseases confirmed by a rheumatologist. Such epidemiological results highlight MSK health issues in marginalized populations and help to include it in the development of detection and treatment programs by the health care authorities. Studies like ours can also help in describing some sociodemographic and functional capacity variables associated with these diseases. Finding these associations can make it easier to plan interventions to prevent and improve disabilities caused by the rheumatic diseases while being sensitive to the sociocultural conditions of the indigenous communities.

We acknowledge that the cross-sectional design of this study prevents the identification of causal inferences. This study was a census of a rural area, and possibly, our results could only be extrapolated to indigenous populations with similar characteristics. Even though the participation rate in this study was 67.3 %, when we compared the sociodemographic characteristics between responders and non-responders, there were no significant differences, implying that selection bias was unlikely.

In conclusion, this study shows that MSK pain and rheumatic diseases are highly prevalent in the Chankom municipality. The most prevalent rheumatic diseases are non-inflammatory (RRPS, OA, low back pain). Older age, being female, disability, and physically demanding work activities were associated with a greater likelihood of having a rheumatic disease. The prevalence of RA was lower than that reported in previous studies in the region. The high impact of rheumatic diseases on daily activities in the indigenous population suggests the need to organize culturally sensitive community interventions for prevention of disabilities caused by MSK disorders and diseases and to design strategies for early detection of rheumatic diseases.

## References

[1] Storheim K, Zwart JA (2014). Musculoskeletal disorders and the global burden of disease study. Ann Rheum Dis.

[2] Chopra A (2013). The COPCORD world musculoskeletal pain and arthritis. Rheumatology.

[3] Abdel-Kader N, Cardiel MH (2011). Geographical factors in rheumatoid arthritis. Int J Clin Rheumatol.

[4] Darmawan J, Muirden KD (2003). WHO-ILAR COPCORD perspectives past, present, and future. J Rheumatol.

[5] Darmawan J (2007). Recommendations from the community oriented program for control of rheumatic disease for data collection for the measurement and monitoring of health in developing countries. Clin Rheumatol.

[6] Muirden KD (2005). Community oriented program for the control of rheumatic diseases: studies of rheumatic diseases in the developing world. Curr Opin Rheumatol.

[7] Montenegro RA, Stephens C (2006). Indigenous health in Latin America and the Caribbean. Lancet.

[8] Fuente: CDI. Sistema de indicadores sobre la población indígena de México con base en: INEGI Censo General de Población y Vivienda, México, 2010. www.cdi.gob.mx/cedulas/2010/YUCA/31017-10. Accessed November 4, 2014.

[9] OECD Reviews of Health Systems. Mexico. Organization for Economic Co-operation and Development. ISBN 92-64-00892-6 – OECD 2005

[10] Peláez-Ballestas I, Sanin LH, Moreno-Montoya J, Alvarez-Nemegyei J, Burgos-Vargas R, Garza-Elizondo M (2011). Epidemiology of the rheumatic diseases in Mexico. A study of 5 regions based on the COPCORD methodology. J Rheumatol Suppl.

[11] Obregon-Ponce A, Iraheta I, Garcıa-Ferrer H, Mejia B, Garcıa-Kutzbach A (2012). Prevalence of musculoskeletal diseases in Guatemala, Central America. The COPCORD study of 2 population. JCR.

[12] Reyes-Llerena GA, Guibert-Toledano M, Penedo-Coello A, Perez-Rodriguez A, Baez-Duenas RM, Charchinaro-Vidal R (2009). Community-based study to estimate prevalence and burden of illness of rheumatic diseases in Cuba: a COPCORD study. J Clin Rheumatol.

[13] Gamboa R, Medina M, Acevedo E, Pastor C, Cucho M, Gutierrez C (2009). Prevalence of rheumatic diseases and disability in an urban marginal Latin American population. A community based study using the COPCORD model. Rev Peru Reumatol.

[14] Granados Y, Cedeño L, Rosillo C, Berbin S, Azocar M, Molina ME I et al (2014) Prevalence of musculoskeletal disorders and rheumatic diseases in an urban community in Monagas State, Venezuela: a COPCORD study. Clin Rheumatol 10.1007/s10067-014-2689-9 24924602

[15] Rodríguez-Senna E, De Barros LP, Silva EO, Costa IF, Pereira LV, Mesquita-Ciconelli R (2004). Prevalence of rheumatic diseases in Brazil: a study using the COPCORD approach. J Rheumatol.

[16] Rodriguez-Amado J, Moreno-Montoya J, Alvarez-Nemegyei J, Goycochea-Robles MV, Sanin LH, Burgos-Vargas R et al (2014) The social gap index and the prevalence of osteoarthritis in the community: a cross-sectional multilevel study in Mexico. Clin Rheumatol 10.1007/s10067-014-2776-y 25227770

[17] Moreno-Montoya J, Alvarez-Nemegyei J, Sanin LH, Pérez-Barbosa L, Trejo-Valdivia B, Santana N, GEEMA (Grupo de Estudio Epidemiológico de Enfermedades Músculo Articulares) (2015). Association of regional and cultural factors with the prevalence of rheumatoid arthritis in the mexican population: a multilevel analysis. J Clin Rheumatol.

[18] Pons-Estel BA, Catoggio LJ, Cardiel MH, Soriano ER, Gentiletti S, Villa AR (2004). The GLADEL multinational Latin American prospective inception cohort of 1,214 patients with systemic lupus erythematosus: ethnic and disease heterogeneity among “Hispanics”. Medicine.

[19] Cardiel MH, Pons-Estel BA, Sacnun MP, Wojdyla D, Saurit V, Marcos JC (2012). Treatment of early rheumatoid arthritis in a multinational inception cohort of Latin American patients: the GLADAR experience. J Clin Rheumatol.

[20] Ferucci ED (2008). Arthritis in indigenous populations: a neglected health disparity. J Rheumatol.

[21] Anaya JM, Rojas-Villarraga A, Mantilla RD, Galarza-Maldonado C (2013) Rheumatoid arthritis in miniorities. Arthritis 256493. doi: 10.1155/2013/256493 10.1155/2013/256493 PMC3666299 23762553

[22] Leyva-Flores R, Infante-Xibille C, Gutierrez JP, Quintino-Pérez F (2013). Inequidad persistente en salud y acceso a los servicios para los pueblos indigenas de México, 2006–2012. Salud Publica Mex.

[23] Pelaez-Ballestas I, Granados Y, Silvestre A, Valls E, Quintana R, Figuera Y (2014). Cross-cultural adaptation of community oriented program for the control of rheumatic diseases methodology in Latin American indigenous population. Rheumatol Int.

[24] Altman R, Alarcón G, Appelrouth D, Bloch D, Borenstein D, Brandt K (1990). The American College of Rheumatology criteria for the classification and reporting of osteoarthritis of the hand. Arthritis Rheum.

[25] Altman R, Asch E, Bloch D, Bole G, Borenstein D, Brandt K (1986). Development of criteria for the classification and reporting of osteoarthritis. Classification of osteoarthritis of the knee. Diagnostic and Therapeutic Criteria Committee of the American Rheumatism Association. Arthritis Rheum.

[26] Arnett FC, Edworthy SM, Bloch DA, McShane DJ, Fries JF, Cooper NS (1988). The American Rheumatism Association 1987 revised criteria for the classification of rheumatoid arthritis. Arthritis Rheum.

[27] Wolfe F, Smythe HA, Yunus MB, Bennett RM, Bombardier C, Goldenberg DL (1990). The American College of Rheumatology 1990 criteria for the classification of fibromyalgia. Report of the Multicenter Criteria Committee. Arthritis Rheum.

[28] Hochberg MC (1997). Updating the American College of Rheumatology revised criteria for the classification of systemic lupus erythematosus [letter]. Arthritis Rheum.

[29] Wallace SL, Robinson H, Masi AT, Decker JL, McCarty DJ, Yu TF (1997). Preliminary criteria for the classification of the acute arthritis of primary gout. Arthritis Rheum.

[30] van der Linden S, Valkenburg HA, Cats A (1984). Evaluation of diagnostic criteria for ankylosing spondylitis. A proposal for modification of the New York criteria. Arthritis Rheum.

[31] Palmer K, Walker-Bone K, Linaker C, Reading I, Kelingray S, Coggon D (2000). The Southampton examination schedule for the diagnosis of musculoskeletal disorders of the upper limb. Ann Rheum Dis.

[32] Alvarez-Nemegyei J, Peláez-Ballestas I, Sanin LH, Cardiel MH, Ramirez-Angulo A, Goycochea-Robles MV (2011). Prevalence of musculoskeletal pain and rheumatic diseases in the Southeastern region of Mexico. A COPCORD-based community survey. J Rheumatol Suppl.

[33] Nicola M, Sawyer S, Parker J, Darmawan J (2004). Rheumatic disease in an Australian aboriginal community in North Queensland. Australia. A WHO-ILAR COPCORD survey. J Rheumatol.

[34] Barnabe C, Elias B, Barlett J, Roos L, Peschken C (2008). Arthritis in aboriginal Manitobans: evidence for a high burden of disease. J Rheumatol.

[35] Ehrlich GE (2003). Back pain. J Rheumatol.

[36] Sim J, Madden S (2008). Illness experience in fibromyalgia syndrome: a metasynthesis of qualitative studies. Soc Sci Med.

[37] Calixto OJ, Anaya JM (2014). Socioeconomic status. The relationship with health and autoimmune diseases. Autoimmun Rev.

[38] Hernández Cáceres AE, Rodriguez Amado J, Peláez Ballestas I, Vega Morales D, Garza Elizondo MA (2014). Factors associated with treatment of osteoarthritis: analysis of a COPCORD study in Nuevo Leon, Mexico. Reumatol Clin.

